# Non-aneurysmal aberrant right subclavian artery causing dysphagia in a young girl: challenges encountered using supraclavicular approach

**DOI:** 10.1186/s13019-015-0303-0

**Published:** 2015-07-05

**Authors:** Ahmad K. Darwazah, Mohammed Eida, Ramzi Abu Khalil, Hassan Ismail, Naser Hanbali

**Affiliations:** 1 Department of Cardiac Surgery, Ramallah Hospital, Ramallah, Israel; 2 Department of Cardiac Anesthesia, Ramallah Hospital, Ramallah, Israel; 3 Makassed Hospital, Mount of Olives, 91194 Jerusalem, Israel

**Keywords:** Congenital vascular anomalies, Anomalies of aortic arch, Aberrant right subclavian artery, Dysphagia lusoria, Arteria lusoria, Supraclavicular approach

## Abstract

Aberrant right subclavian artery is the most common anomaly of the aortic arch. Patients are often asymptomatic and discovered accidentally. Occasionally, they present with symptoms related to oesophageal or tracheal compression.

A 13-year-old girl presented with dysphagia and stridor was found to have an aberrant right subclavian artery. Surgical division and reconstruction of the artery was performed initially through right supraclavicular approach. An additional left thoracotomy was performed to overcome the challenges encountered at initial operation.

## Introduction

Aberrant right subclavian artery (ARSA) or arteria Lusoria is the most common of relatively uncommon congenital vascular anomaly of the aortic arch. It results from disruption of remodelling of branchial arches of the right dorsal aorta distal to the sixth cervical intersegmental artery [1].

Most patients are asymptomatic. Rarely, it causes dysphagia (dysphagia lusoria) and respiratory symptoms, which usually present in the fourth or fifth decades [2].

A symptomatic young girl with ARSA is presented. Surgical management and the difficulties encountered during operation are discussed.

## Review

A 13-year-old girl was admitted for evaluation of progressive dysphagia associated with shortness of breath. Her symptoms dated back to when she was 5 years of age, when her mother noticed that she could only eat small, frequent soft meals due to difficulty of swallowing.

The patient was seen on several occasions by dieticians and endocrinologist, who assured the family that their daughter is completely normal. Gradually, the condition progressed and was associated with intermittent sudden attacks of stridor just one month before admission.

**Fig. 1:**
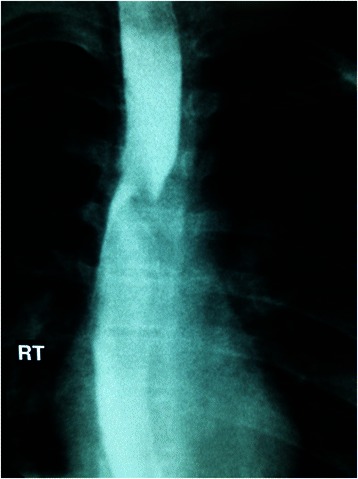
Barium swallow showing an oblique posterior indentation of the oesophagus

**Fig. 2:**
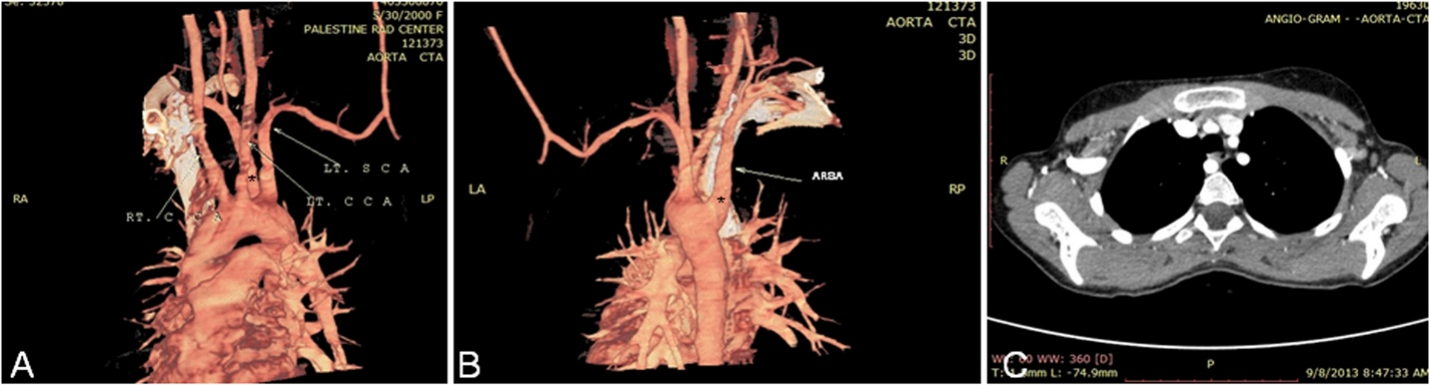
**a** Four branches arises from the aortic arch. Aberrant right subclavian artery(ARSA) marked by astrex. **b** ARSA arises distal to left subclavian artery. **c** Obstruction of the oesophagus posteriorly by ARSA

Physical examination revealed a thin, otherwise healthy, young girl. Laboratory tests, chest rentegenography, echocardiography and abdominal CT were normal. However, Barium swallow showed an oblique posterior indentation of the oesophagus just above the level of aortic arch (Fig. 1). CT angiography revealed a left aortic arch with an aberrant right subclavian artery originating from the aorta distal to left subclavian artery (Fig. 2a,b). The artery was crossing the midline compressing the posterior surface of the oesophagus (Fig. 2c).

The patient underwent surgical reconstruction using right supraclavicular approach. The right common carotid artery, right internal Jugular vein and the aberrant right subclavian artery were exposed, after division of clavicular head of sternomastoid and scalenous anterior muscles.

**Fig. 3:**
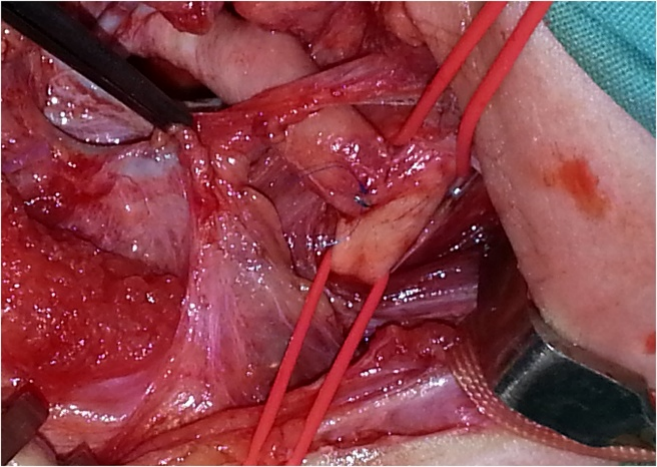
Intraoperatively showing anastomosis of ARSA to the side of right common carotid artery

The aberrant artery was dissected along its retroesophageal route. During dissection, injury of an aberrant right thoracic duct occurred and was repaired. The right vertebral artery was identified and controlled. Exposure of the origin of the aberrant artery was facilitated by gentle traction of the artery during dissection and by displacement of both trachea and oesophagus. Once the origin was identified, it was doubly ligated near the aortic arch. The proximal segment was anastomosed end to side to the right common carotid artery (Fig. 3).

Finally, the patient was extubated with normal vocal cord function and equal pulse and blood pressure on both sides.

**Fig. 4:**
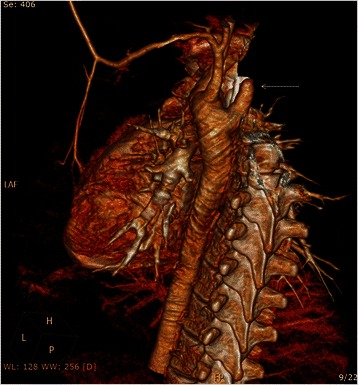
Postoperative CT angiography showing a remaining stump of the origin of ARSA

Postoperatively, the patient was able to eat normally on the 2^nd^ day. Ptosis and anisocoria were observed in the right eye. Oral corticosteroids were given after normal ophthalmological examination. Just before discharge a CT angiography was performed. It showed a long stump of the remaining part of the aberrant artery at its origin (Fig. 4).

**Fig. 5:**
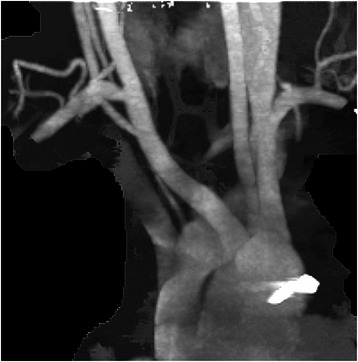
Follow up CT angiography showing complete ligation of the origin of ARSA and patent anastomosis of ARSA to right common carotid artery

A second operation was performed though left antero-lateral thoracotomy in the 3^rd^ intercosal space. The ARSA appeared dilated originating from the proximal part of the descending aorta. It was ligated, and the stump was sutured by continuous prolene . The patient was discharged after 3 days in good condition. On follow up, the patient remained asymptomatic and was eating normally. CT angiography showed absence of the stump with patent anastomotic site (Fig. 5).

## Discussion

Aberrant right subclavian artery (ARSA) is the most common anomaly of the aortic arch. Its incidence varies from 0.5 to 2.5 % [1]. The artery originates on the left side as a last branch of the aortic arch beyond the left subclavian artery. It follows an oblique course, crossing upward and to the right behind the oesophagus. Rarely, it may cross between the oesophagus and trachea or infront of the trachea [1].

ARSA may occur as an isolated lesion, but in the majority of cases an associated aortic arch anomalies may occur. For example truncus bicaroticus, abnormal origin of right vertebral artery, or right inferior thyroid artery arise from right common carotid and aortic coarctation may occur [3].

In 68 % of cases, associated cardiac anomalies in the form of septal defects, left- sided cardiac lesions and conotruncal anomalies may be present [4].

The majority of patients with ARSA are asymptomatic. Such an anomaly is usually discovered accidentally.

The close relationship between the ARSA, oesophagus and trachea is responsible for various presentations seen among these patients. Compression of oesophagus and trachea may cause dysphagia, dyspnea or pneumonia [5]. Such symptoms worsen with age when compression becomes more pronounced as oesophageal rigidity and atherosclerotic hardening of arteries increase. Rarely, aneurismal dilatation may occur, causing right arm ischemia, thoracic outlet syndrome, oesophageal fistulization and rupture [2].

ARSA may be diagnosed by performing chest roentgenography [6]. Oesophagography can provide a more accurate diagnosis where an oblique defect or indentation along the posterior aspect of the oesophagus can be seen extending from left to right just above aortic arch [1]. A CT or MR angiography will offer a definite visualization of arch anatomy and ARSA.

Our young patient had an isolated ARSA which presented with genuine symptoms related to oesophageal compression. She had a typical dysphagia to solid food and adapted by decreasing food intake or eating small frequent meals with plenty of fluids. This led to a delay in the diagnosis for almost 8 years. The appearance of respiratory symptoms in the form of intermittent attacks of stridor, which were presumably caused by stagnation of food in the oesophagus and weight loss, triggered further investigations.

The majority of patients with ARSA are asymptomatic and require no further treatment. To avoid further complications, patients with symptoms related to oesophageal or tracheal compression and those with aneurismal dilatation, need surgical intervention.

Primarily, surgery will ligate the aberrant artery followed by restoration of blood flow to the right arm by anastomosing the ARSA to either the right common carotid artery, aortic arch or ascending aorta, with or without interposition graft [7]. Failure to ligate the artery close to its origin may lead to residual dysphagia [8]. Simple ligation and division without restoring blood flow to the right arm may cause subclavian steal syndrome, arm ischemia and gangrene [9].

This can be achieved by a variety of surgical approaches including median sternotomy, left thoracotomy, right supraclavicular approach, combined left thoracotomy and right supraclavicular approach or right thoracotomy [10]. Each technique has its limitations and potential complications.

In the present case, a right supraclavicular approach was used, to provide exposure with a single incision. This approach was used in previous studies [2, 10–13], with satisfactory results and minimal morbidity. However, this approach requires dissection in a limited area especially behind the oesophagus and near the origin of the aberrant artery, which is a deeply situated structure that is difficult to reach. To overcome these difficulties, dissection can be facilitated with the aid of cervical mediastinoscopy that was found highly effective, as indicated by previous studies [12, 14]. Other investigators [11, 15] successfully used a hybrid endovascular approach. Here the origin of the artery was occluded by using Amplatzer plug, followed by anastomosing the divided ARSA to the right common carotid through supraclavicular approach.

Despite meticulous dissection in our patient, various challenges were encountered. An associated aberrant right thoracic duct was recognised after being injured and repaired. To avoid unnecessary morbidity, recognition and immediate repair is important [3, 16].

Postoperatively, our patient had atypical form of Horner’s syndrome. She had ptosis and anisocoria with no evidence of anhidrosis. The close proximity of the preganglionic fibers of the oculosympathetic pathway to the supraclavicular area makes it vulnerable to injury. Traction of neurovascular structures including brachial plexus, right subclavian and carotid artery during dissection can distort the ventral roots and interrupts sympathetic innervation causing Horner’s syndrome. Fortunately, these changes were temporary and subsided completely after 3 weeks with conservative treatment.

Although the patient had complete resolution of symptoms, a residual stump at the origin of the artery was found. Failure to ligate the artery as close as possible to its origin for two reasons. First, the base of the artery was broad, which gave a false impression of reaching the aortic arch during surgery. Second, the artery in this case originated from the proximal part of the descending aorta made it difficult to ligate at its origin. Despite the fact that the residual stump was causing no obstruction, resection through a second operation was performed to avoid possible aneurismal formation in the future.

## Conclusions

Surgical management of ARSA in children using supraclavicular approach can be challenging. To avoid unnecessary complications, a combined left thoracotomy can be used to ligate the origin of ARSA especially when it has a wide base originating from the proximal descending aorta.

## Consent

Written informed consent was obtained from the patients family for publication of this case report and any accompanying images. A copy of the written consent is available for review by the Editor-in-Chief of this journal.
